# Leaf Segmentation Using Modified YOLOv8-Seg Models

**DOI:** 10.3390/life14060780

**Published:** 2024-06-20

**Authors:** Peng Wang, Hong Deng, Jiaxu Guo, Siqi Ji, Dan Meng, Jun Bao, Peng Zuo

**Affiliations:** 1 College of Arts and Sciences, Northeast Agricultural University, Harbin 150030, China; pengwang@neau.edu.cn (P.W.); cshdeng@neau.edu.cn (H.D.); a20220200@neau.edu.cn (S.J.); a02200303@neau.edu.cn (D.M.); 2 College of Animal Science and Technology, Northeast Agricultural University, Harbin 150030, China; jbao@neau.edu.cn; 3 National Key Laboratory of Smart Farm Technology and System, Harbin 150030, China; 4 College of Life Science, Northeast Agricultural University, Harbin 150030, China; a09210131@neau.edu.cn

**Keywords:** leaf segmentation, yolo, computer-vision

## Abstract

Computer-vision-based plant leaf segmentation technology is of great significance for plant classification, monitoring of plant growth, precision agriculture, and other scientific research. In this paper, the YOLOv8-seg model was used for the automated segmentation of individual leaves in images. In order to improve the segmentation performance, we further introduced a Ghost module and a Bidirectional Feature Pyramid Network (BiFPN) module into the standard Yolov8 model and proposed two modified versions. The Ghost module can generate several intrinsic feature maps with cheap transformation operations, and the BiFPN module can fuse multi-scale features to improve the segmentation performance of small leaves. The experiment results show that Yolov8 performs well in the leaf segmentation task, and the Ghost module and BiFPN module can further improve the performance. Our proposed approach achieves a 86.4% leaf segmentation score (best Dice) over all five test datasets of the Computer Vision Problems in Plant Phenotyping (CVPPP) Leaf Segmentation Challenge, outperforming other reported approaches.

## 1. Introduction

The segmentation of individual leaves of a plant is a prerequisite for measuring more complex phenotypic traits such as shape, color, area, mass, or texture, or for counting the number of leaves. For instance, biologists cultivate model plants like Arabidopsis (*Arabidopsis thaliana*) and tobacco (*Nicotiana tabacum*) in controlled environments, monitoring and documenting their phenotypes to investigate the performance of plants in general. Previously, such phenotypes were annotated manually by experts, but recently, image-based non-destructive approaches have gained attention among plant researchers in plant classification, monitoring of plant growth, precision agriculture, and other scientific research [1].

Leaf segmentation can be divided into two categories: one is isolated leaf segmentation and identification, and the other is live plant leaf segmentation. The second category can further be divided into two sub-categories, leaf semantic segmentation and leaf instance segmentation. 

Isolated leaf segmentation and identification usually uses datasets with leaves that have been cut from plants and imaged individually. Leaf classification and disease identification are the most common tasks in this category. Françoise used a binary thresholding technique to segment the leaf and extract leaf texture to classify plants into families [2]. Shoaib used modified U-Net to segment the tomato leaf and then Inception Net to classify the segmented images by different levels of disease [3]. For this task, in most cases there is only one leaf in the image. Some leaf classification approaches can even be done without the segmentation stage. For example, Bin Wang used a parallel two-way convolutional neural network to classify the leaf category in Flavia [4], Swedish [5], and Leafsnap [6] datasets and achieved above 91% performance in all three datasets [7]. Various types of deep convolution networks were employed in leaf classification tasks such as lightweight CNN [8], Siamese network [9], and ResNet [10,11]. These works show that for isolated leaf segmentation and identification, if the images are taken carefully with only one leaf in the center and a clear background, researchers can achieve very good performance in their tasks even if they omit the segmentation or detection stage.

Live plant leaf segmentation is another category and has many differences from isolated leaf segmentation because live plant leaf segmentation is to segment the leaves of the plant in the image, while isolated leaf segmentation is to segment the leaves that have been cut from the plant and placed on a plain background. The typical difficulties of live plant leaf segmentation include: (1) The live plant usually has multiple leaves. (2) The leaves are shot from different angles, so they have different shapes, poses, and appearances. (3) The background may not be very clear. (4) It is hard to find clearly discernible boundaries among overlapping leaves. Thus, live plant leaf segmentation usually requires more complex algorithms and models to handle the complex background and lighting conditions in the image, as well as to handle the deformation and overlapping of leaves.

Live plant leaf segmentation involves two different tasks, leaf semantic segmentation and leaf instance segmentation. The difference between semantic segmentation and instance segmentation lies in the objects they segment. Semantic segmentation classifies each pixel into a category (such as leaves or background), while instance segmentation divides each object into a separate entity (such as a single leaf).

Leaf semantic segmentation is usually required by precision agriculture, agricultural robotics, and weed identification. Andres Milioto presented a CNN-based semantic segmentation approach for crop fields, separating sugar beet plants, weeds, and background based on RGB data in real-time [12]. Tanmay Anand proposed a deep learning framework, AgriSegNet, for automatic detection of farmland anomalies using multiscale attention semantic segmentation of UAV-acquired images [13]. Sovi Guillaume Sodjinou used U-Net and K-means subtractive algorithm to apply the semantic segmentation of crops and weeds [14].

Many studies leverage leaf instance segmentation, including growth monitoring and regulation, and counting [15,16]. In one study, Bhugra et al. proposed a framework that relies on a graph-based formulation to extract leaf shape knowledge for the task of leaf instance segmentation [17]. Numerous creative approaches employ varied types of mathematics, modeling and computer science approaches including 3D data augmentation [18], generative adversarial networks, and Mask R-CNN [19].

In this work, we put our focus on the problem of leaf instance segmentation and proposed two improved versions of the YOLOv8-seg model for automatic segmentation of individual leaves in images. To enhance the segmentation performance, we introduce a Ghost module and a BiFPN module to the standard YOLOv8 model. The Ghost module can generate multiple intrinsic feature maps through several inexpensive transformation operations, while the BiFPN module can fuse multi-scale features to improve the segmentation performance of small leaves. We also confirmed through ablation experiments that the BiFPN and Ghost module do help to improve the classification effect to a certain extent.

## 2. Materials and Methods

### 2.1. Datasets

**Figure 1:**
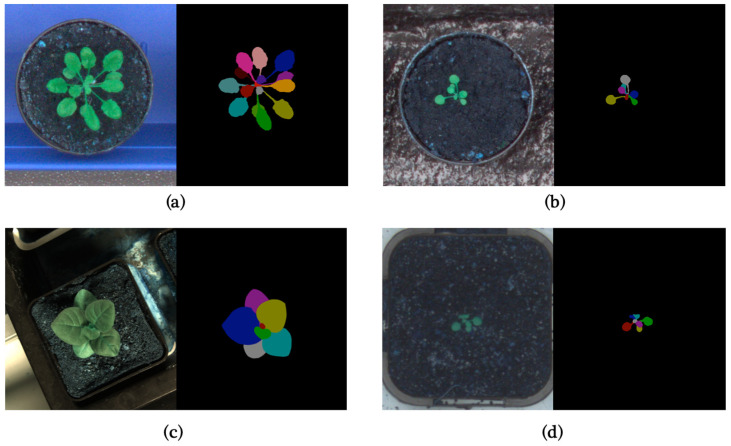
(**a**) Typical image in A1; (**b**) Typical image in A2; (**c**) Typical image in A3; (**d**) Typical image in A4.

In this paper, to make the results as comparable as possible, we use the CVPPP LSC dataset [1,20,21] for training and validating our model. It is one of the most significant datasets in this field and has almost become a benchmark for these tasks. The CVPPP LSC dataset was presented in the Leaf Segmentation Challenge of Computer Vision Problems in Plant Phenotyping Workshop, which is also the origin of the name CVPPP LSC. The LSC 2014 training dataset includes three subsets (A1, A2, A3), with A1 and A2 consisting of 159 time-lapse images of Arabidopsis and A3 consisting of 27 images of tobacco. In LSC 2017, a new subset A4 was introduced, consisting of 624 images of Arabidopsis shared by Dr. Hannah Dee from Aberystwyth [21]. Figure 1 shows typical images from different datasets, and Table 1 is a brief summary of the LSC training dataset.

The LSC testing set can be divided into two groups. The first group is A1–A4, which corresponds to the training set of A1–A4 respectively. The second group is A5, which is basically a combination of the data of A1–A4. Table 2 is a brief summary of the LSC testing dataset. CVPPP did not release the ground truth of the testing set; to evaluate the performance of the testing set, the results need to be uploaded to the competition website (https://codalab.lisn.upsaclay.fr/competitions/8970, accessed on 1 January 2024) for online calculation, and the performance of the testing set needs to be uploaded and evaluated via the competition site.

### 2.2. Framework

**Figure 2:**
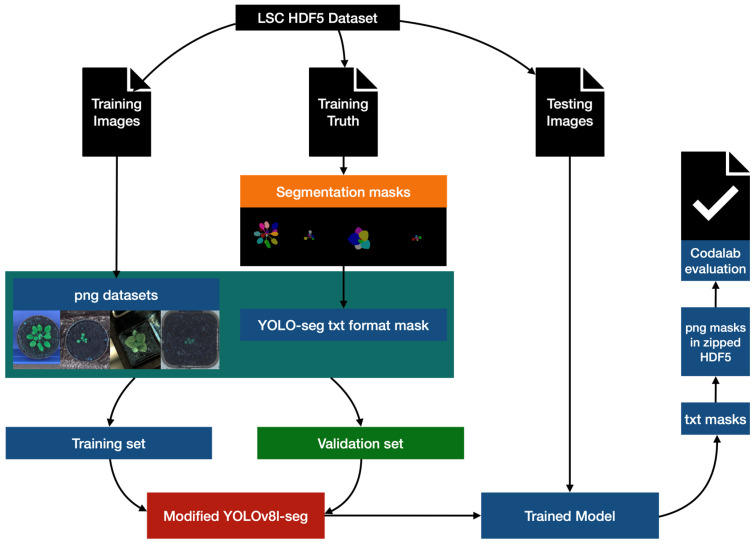
The working process of data processing, model training, and model validation.

The working process of data processing, model training, and model validation is shown in Figure 2. It was not always the case that datasets were provided in a format which was compatible with YOLOv8. In the preprocessing, we employ the h5py and OpenCV to appropriately transform the dataset into png format with txt masks [22,23]. To prevent overfitting, we have created a training set that is 3/4 the size of the original training set and a validation set that is 1/4 the size by taking the fourth one after every three in the file list. Then we develop a modified YOLOv8-seg model and train the model with the split training and validation sets, and use it to segment the official test set. We incorporate the BiFPN module (Bidirectional Feature Pyramid Network) [24] and Ghost module [25] into the original YOLOv8-seg model to enhance the segmentation performance. The segmentation results were submitted to the competition website for performance evaluation. 

### 2.3. Methods

In this work, the YOLOv8 model and two modified versions were used for leaf segmentation. YOLO introduced a new, simplified way to perform simultaneous object detection and classification in images [26,27]. The latest version of YOLO is Yolov8, released in January 2023 by Ultralytics [28], who also created the earlier version YOLOv5 [29]. YOLOv8 uses techniques similar to YOLOACT [30] to provide support for instance segmentation. 

**Figure 3:**
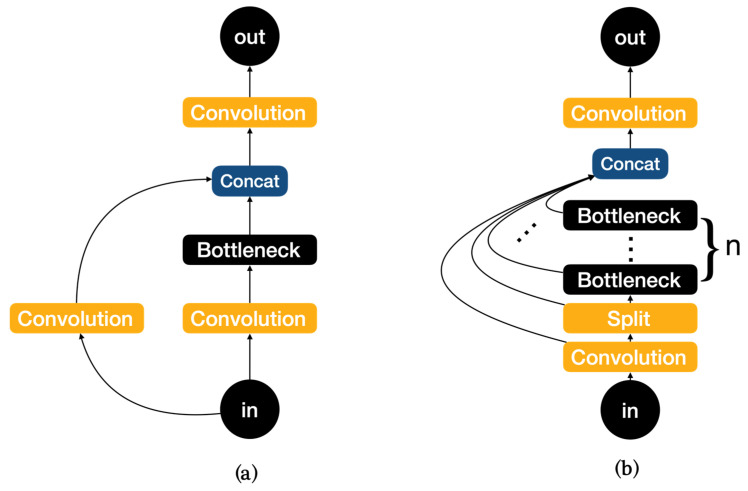
(**a**)Architecture of C3. (**b**) Architecture of C2f.

The network structure mainly consists of backbone, neck, and head, with YOLOv8 replacing the C3 module of YOLOv5 with the C2f module in the backbone as shown in Figure 3. It is easy to see that the C2f module has a richer gradient flow. The head section adopts a popular decoupled head structure which separates the classification and detection heads, and also is converted from anchor-based to anchor-free, reducing the number of box predictions and speeding up the non-maximum impression (NMS). In loss calculation, Task Aligned Assigner is used for positive sample allocation and Distribution Focal Loss is introduced. The data augmentation section incorporates the operation of closing Mosaic enhancement in the last 10 epochs of YOLOX [31], which can effectively improve accuracy.

**Figure 4:**
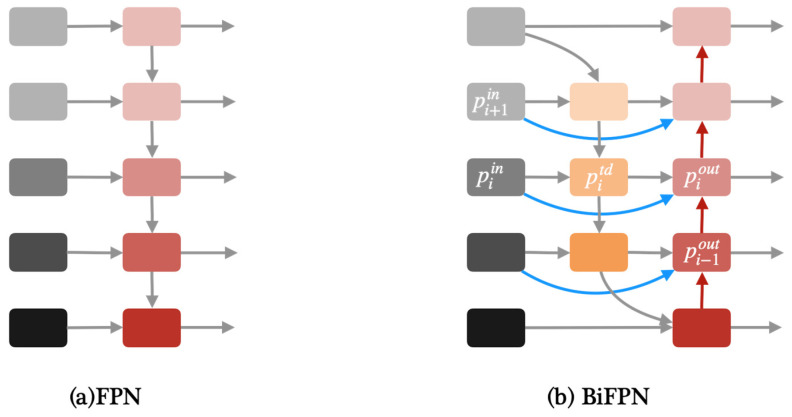
(**a**)Architecture of FPN. (**b**) Architecture of BiFPN.

Apart from YOLOv8, we proposed two modified YOLOv8 models in this paper, one of which is YOLOv8-BiFPN. FPN (Feature Pyramid Network) is used to solve the problem of multi-scale in object detection, which can improve detection performance with small targets [32]. Compared with the traditional FPN network, BiFPN adds skip connections between the input and output features in the same layer [24]. Due to using the same scale, adding skip connections can better extract and transfer feature information. Figure 4 shows the architecture of FPN and BiFPN.

BiFPN uses weighted feature fusion to fuse input feature maps of different resolutions. The weights in BiFPN were calculated as follows:

(1) $$P_{i}^{td}=Convolution(\frac{w_{1}\cdot P_{i}^{in}+w_{2}{\cdot P}_{i+1}^{in}}{w_{1}+w_{2}+\epsilon }),$$

(2) $$P_{i}^{out}=Convolution(\frac{w_{1}^{\prime }{\cdot P}_{i}^{in}+w_{2}^{\prime }{\cdot P}_{i}^{td}+w_{3}^{\prime }{\cdot P}_{i-1}^{out}}{w_{1}^{\prime }+w_{2}^{\prime }+w_{3}^{\prime }+\epsilon }),$$

where $P_{i}^{td}$, $P_{i}^{out}$ represent the intermediate transition feature of the *i*-th layer on the top-down pathway and the last output feature of the *i*-th layer on the bottom-up pathway. $w_{1}$, $w_{2}$ are the weight parameters of the input of the current layer and the input of the next layer. In Formula (2), $w_{1}^{\prime }$, $w_{2}^{\prime }$, $w_{3}^{\prime }$ respectively represent weight of the current layer input, the weight of the transition unit output in the current layer, and the weight of the previous layer output. *ϵ* is a hyperparameter to prevent the gradient from vanishing [24]. 

**Figure 5:**
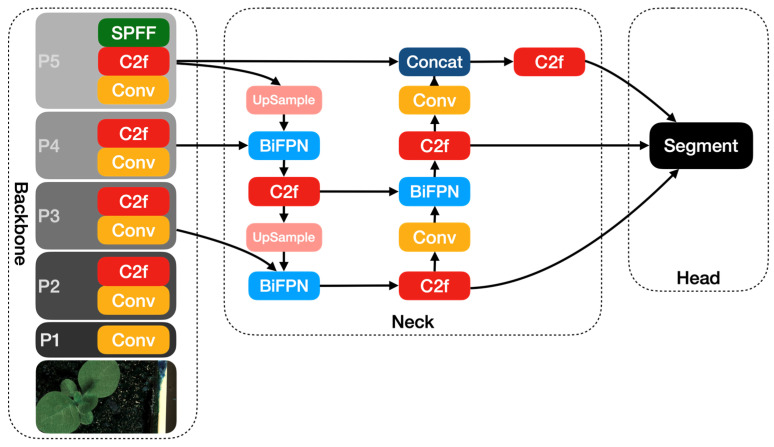
The YOLOv8-BiFPN network architecture diagram.

In this paper, we incorporate the BiFPN module into the neck of the YOLOv8 module and present the YOLOv8-BiFPN model. Figure 5 shows the architecture of the YOLOv8-BiFPN model.

**Figure 6:**
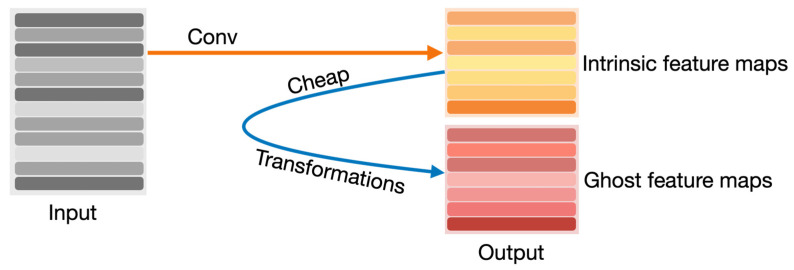
Principle of Ghost module.

**Figure 7:**
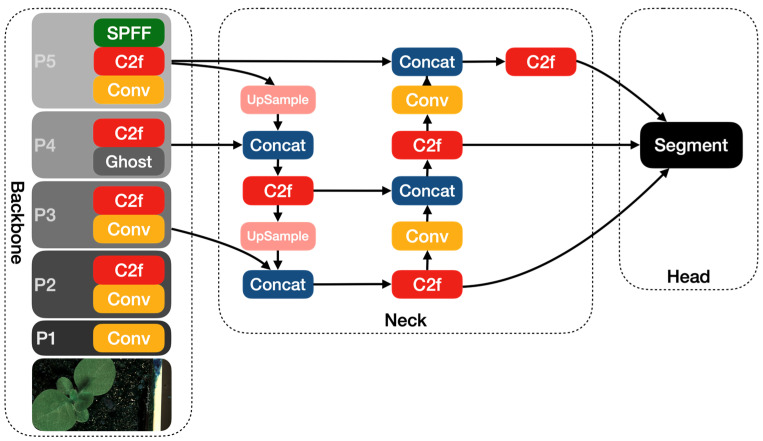
The YOLOv8-Ghost network architecture diagram.

The other modified YOLOv8 model that we propose in this article is YOLOv8-Ghost. The Ghost module splits the original convolutional layer into two parts and utilizes fewer filters to generate several intrinsic feature maps. Then, a certain number of cheap transformation operations will be further applied for generating Ghost feature maps efficiently [25]. Figure 6 shows the principle of the Ghost module. The Ghost module includes two sets of feature maps: the intrinsic feature, which is composed of the convolution of the input, and another set which is composed of some cheap transformation results of the first set (in this work the cheap transformation refers to the 5 × 5 convolution). Thus, the Ghost module can reduce the computational effort and generate richer feature maps, which is helpful in enhancing the generalizing ability of the model. We incorporate the Ghost module into the backbone of the YOLOv8 model and present the YOLOv8-Ghost model. Figure 7 presents the YOLOv8-Ghost network architecture. It consists of backbone, neck, and head; the backbone is a Feature Pyramid Network [32] to deal with the multi-scale issue and the neck is a Path Aggregation Network [33] to boost the information flow. From Figure 7, one can find that the convolution block in P4 of the backbone was substituted with the Ghost module to boost the generalizing ability.

### 2.4. Data Augmentation

Data augmentation is a technique commonly used in machine learning to increase the size and diversity of a dataset. It involves applying various transformations to the existing data to create new, synthetic samples. In this paper, we apply some data augmentations supported by YOLOv8, including adjustment of the HSV color, translating the image horizontally and vertically by a fraction, scaling the image by a gain factor to simulate objects at different distances, flipping the image upside down and left to right with the specified probability, rotating the image randomly within the specified degree range, combining four training images into one to simulate different scene compositions, and randomly erasing a portion of the image during classification training. The specific parameters for data augmentation are shown in Table 3. 

### 2.5. Software and Hardware Environments

The experiments were conducted on a computer with Nvidia Tesla P100 GPU. The detailed information of software and hardware environments are listed in Table 4.

## 3. Results

### 3.1. Evaluation Metrics

To evaluate the performance of multi-object segmentation, the five metrics used in the CVPPP Leaf Segmentation Challenge were adopted for result comparison [34], i.e., the Best Dice (BD) [35,36], Symmetric Best Dice (SBD), Foreground–Background Dice (FGBGDice), Difference in Count (DiffFG), and Absolute Difference in Count (AbsDiffFG). 

Dice is a metric in binary segmentation that measures the degree of overlap between the ground truth $L^{gt}$ and the algorithmic result $L^{ar}$, as defined in Equation (3).

(3) $$Dice\left(L^{ar},L^{gt}\right)=\frac{2\left|L^{gt}\cap L^{ar}\right|}{{|L}^{gt}|+|L^{ar}|}$$

Best Dice is defined as:

(4) BDLar,Lgt=1M∑i=1Mmax1≤j≤N2Ligt∩LiarLigt+Liar

Symmetric Best Dice is the symmetric average Dice among all leaves, which is defined as:

(5) $$SBD=\min\{BD\left(L^{ar},L^{gt}\right),BD(L^{gt},L^{ar})\}$$

Foreground–Background Dice (FGBGDice) is the Dice score of the foreground mask (i.e., masks of all leaves).

Difference in Count (DiffFG) is a metric used to evaluate how well an algorithm identifies the correct number of leaves present, and is defined as:

(6) $$DiffFG={\#L}^{ar}-\#L^{gt}$$

Absolute Difference in Count (AbsDiffFG) is the absolute difference in object count.

(7) $$AbsDiffFG=|{\#L}^{ar}-{\#L}^{gt}|$$

### 3.2. Segmentation Results

**Figure 8:**
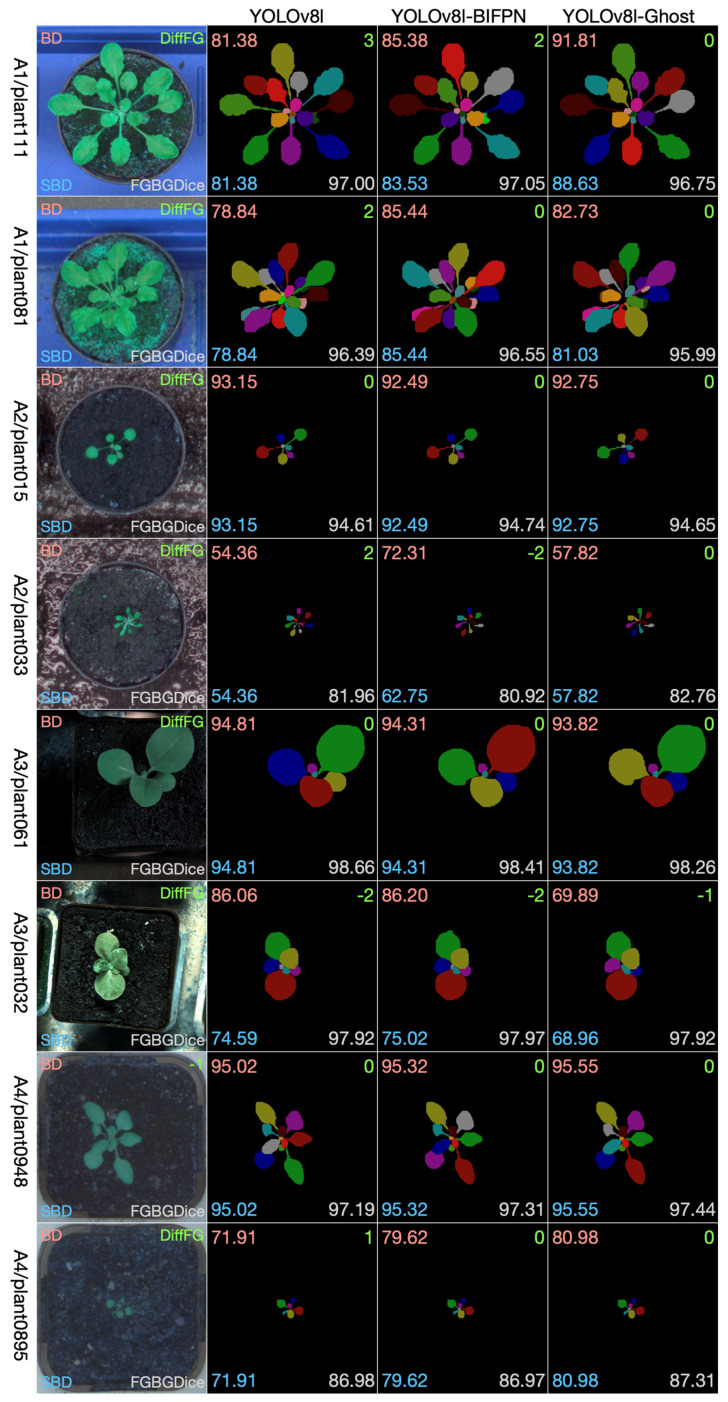
Selected results on test images from each dataset.

The leaf instance segmentation on the CVPPP LSC dataset was performed by the YOLOv8-seg and the two modified versions, namely the YOLOv8-BiFPN and YOLOv8-Ghost. Taking into comprehensive consideration the data size, the GPU memory size, and the performance, we chose the l-scale in the scale selection of the three models. During the training stage, all of the training sets (A1, A2, A3, A4) were combined together to create a larger dataset; 3/4 was used for training, and 1/4 was used for validation. Since the original size of the dataset is close to 512 × 512, all of the images were resized to 512 × 512. The neural network was trained through 250 epochs with batch size 16. Figure 8 shows selected examples of test images from the four datasets. From each dataset we chose two examples: one to show the effectiveness of the methods and one to show limitations. We have four datasets, so eight pictures were selected and composed into the eight rows in Figure 8. The first column is the test leaf image, the second column is the segmentation result using YOLOv8, the third column is the segmentation result using YOLOv8-BiFPN, and the fourth column is the segmentation result using YOLOv8-Ghost. We show visually the segmentation outcomes for each method together and overlay the numbers of the evaluation measures on the images (Best Dice in the top left corner, Difference in Count in the top right, Symmetric Best Dice in the bottom left, and Foreground–Background Dice in the bottom right).

For these eight test data examples, we see that all of the three methods perform well in the segmentation task. There is not a certain method that is absolutely better than the other two methods. It can be observed that YOLOv8-Ghost is more precise in the counting of the number of leaves, but in other indicators, the three methods have their own advantages and disadvantages.

The segmentation result for the whole test dataset is shown in Table 5. It can be seen that among the three methods, the performance of the two modified versions is slightly better than that of the original YOLOv8. The performance of YOLOv8-BiFPN is slightly better in SBD and FGBGDice, while the performance of YOLOv8-Ghost is slightly better in BD, SBD, DiffFG, and AbsDiffFG than that of the other two methods.

We also compared our results with other reported works, as shown in Table 6. Because the results for test sets A5 and A6 were not reported in their papers, only the results for test sets A1, A2, and A3 were compared. From Table 6, one can see that the proposed YOLOv8-BiFPN and YOLOv8-Ghost outperform other approaches with respect to many indicators.

In order to compare with the algorithms of other contestants, we submitted the segmentation results of YOLOv8-Ghost to the competition’s leaderboard, as it has demonstrated stronger performance on Best Dice than YOLOv8-BiFPN, and the competition website mainly uses Best Dice to determine the ranking. Our algorithm’s average ranking on the leaderboard (https://codalab.lisn.upsaclay.fr/competitions/8970#results, accessed on 20 April 2024) is third place as of writing this manuscript (20 April 2024) (our name is “pw” on the leaderboard), and the specific rankings of different test sets by different metrics are shown in Table 7.

## 4. Discussion

In the experiment, we found that the YOLOv8-based [28] methods outperform other approaches in the leaf segmentation task. Moreover, we present two modified versions, i.e., the YOLOv8-BiFPN and YOLOv8-Ghost, which can further improve the performance.

We believe that the BiFPN module [24] can add skip connections between the input and output features in the same layer, which result in better extract and transfer feature information. The Ghost module [25] can reduce the computational effort and generate richer feature maps, which is helpful in enhancing the generalizing ability of the model. Readers may think that the combination of the BiFPN and Ghost modules might further improve the performance; in fact, we conducted the corresponding experiments, but unfortunately, the results were not ideal. Only a few indicators were improved, and most of the indicators were not as positively affected as using one kind of improvement alone. This may be because the BiFPN module tends to increase the complexity on the basis of YOLOv8, while the Ghost module tends to reduce the computational complexity. In the process of combination of the BiFPN and Ghost module, the two modules may mutually influence each other, and the result is not ideal.

It can be found that there is a large number of defocused blurs in the dataset. Some corrections can be made for these defocused blurs in the pre-processing in future work. In addition, although the focus of this article is the instance segmentation of the leaves of living plants, these plants were all planted in flower pots. The leaf segmentation of plants in the fields undoubtedly is more challenging, and it is also a direction worthy of further research.

## 5. Conclusions

In this paper, we presented and evaluated a framework for leaf instance segmentation. The YOLOv8 model was employed for the leaf instance segmentation task; moreover, we proposed two modified versions by incorporating the BiFPN module and Ghost module into the original YOLOv8 model to enhance the leaf segmentation performance. In the experiment, we found that the three methods outperform other approaches in the leaf segmentation task, and the proposed YOLOv8-BiFPN and YOLOv8-Ghost can further improve the performance. YOLOv8-BiFPN shows better performance in FGBGDice, which is used to separate the leaf from the background, and YOLOv8-Ghost shows better performance in BestDice and leaf counting metrics like DiffFG and AbsDiffFG.

## Figures and Tables

**Table 1:** Summary of the LSC training dataset.

Dataset	Plant	Size	Resolution
A1	Arabidopsis	128	530 × 500
A2	Arabidopsis	31	530 × 565
A3	Tobacco	27	2448 × 2048
A4	Arabidopsis	624	441 × 441

**Table 2:** Summary of the LSC testing dataset.

Dataset	Plant	Size	Resolution
A1	Arabidopsis	33	530 × 500
A2	Arabidopsis	9	530 × 565
A3	Tobacco	56	2448 × 2048
A4	Arabidopsis	168	441 × 441
A5	Arabidopsis + Tobacco	235	various

**Table 3:** Data augmentation parameters.

Argument	Value	Short Description
hsv_h	0.015	Adjusts the hue of the image by a fraction of the color wheel
hsv_s	0.7	Alters the saturation of the image by a fraction
hsv_v	0.4	Modifies the value (brightness) of the image by a fraction
degrees	180	Rotates the image randomly within the specified degree range
translate	0.1	Translates the image horizontally and vertically by a fraction
scale	0.5	Scales the image by a gain factor
flipud	0.5	Flips the image upside down with the specified probability
fliplr	0.5	Flips the image left to right with the specified probability
mosaic	1	Combines four training images into one
erasing	0.4	Randomly erases a portion of the image during classification training

**Table 4:** Summary of software and hardware environments.

Hardware		Software	
CPU:	Intel^®^ Core™ i5-8600K	OS:	Windows 10 (21H2)
RAM:	Corsair^®^ 32 GB DDR4	CUDA:	11.8
GPU:	NVIDIA^®^ Tesla^®^ P100 16G	Python:	3.8
		PyTorch:	2.2.0
		torchvision:	0.17.0
		Ultralytics:	8.0.228

**Table 5:** Segmentation and counting results on the testing dataset.

Test Set	BD	SBD	FGBGDice	DiffFG	AbsDiffFG
YOLOv8					
A1	82.71	82.19	96.43	1.67	1.91
A2	82.89	82.98	93.87	1.33	1.33
A3	85.85	71.52	92.61	−1.13	1.34
A4	85.23	84.34	**95.47**	1.12	1.33
A5	85.45	81.37	94.78	0.55	1.30
ALL	85.19	81.35	94.86	0.64	1.36
YOLOv8-BiFPN					
A1	83.08	82.60	96.43	1.52	1.70
A2	83.62	81.82	93.51	1.44	2.11
A3	85.53	**71.53**	**93.55**	−**0.89**	**1.32**
A4	85.77	84.87	95.45	1.01	1.23
A5	85.72	**81.69**	**94.98**	0.54	1.24
ALL	85.50	**81.68**	**95.05**	0.62	1.24
YOLOv8-Ghost					
A1	**85.18**	**83.67**	**96.46**	**0.67**	**1.21**
A2	**84.03**	**83.30**	**93.89**	**0.78**	**1.00**
A3	**86.11**	69.49	92.00	−1.18	1.43
A4	**86.58**	**85.37**	95.44	**0.79**	**1.11**
A5	**86.52**	81.60	94.61	**0.29**	**1.17**
ALL	**86.36**	**81.68**	94.70	**0.32**	**1.18**

The result in bold font means that the result is superior to others.

**Table 6:** Segmentation and counting results compared with other published methods.

Test Set	SBD	FGBGDice	DiffFG	AbsDiffFG
IPK [37]				
A1	74.4	**97.0**	−1.8	2.2
A2	76.9	**96.3**	−1.0	1.2
A3	53.3	94.1	−2.0	2.8
Nottingham [1]				
A1	68.3	95.3	−3.5	3.8
A2	71.3	93.0	−1.9	1.9
A3	51.6	90.7	−1.9	2.5
MSU [16]				
A1	66.7	94.0	−2.5	2.5
A2	66.6	87.7	−2.0	2.0
A3	59.2	90.7	−2.3	2.3
Wageningen [1]				
A1	71.1	94.7	1.3	2.2
A2	75.7	95.1	−**0.2**	**0.4**
A3	57.6	89.5	1.8	3.0
YOLOv8				
A1	82.19	96.43	1.67	1.91
A2	82.98	93.87	1.33	1.33
A3	71.52	92.61	−1.13	1.34
YOLOv8-BiFPN				
A1	82.60	96.43	1.52	1.70
A2	81.82	93.51	1.44	2.11
A3	**71.53**	**93.55**	−**0.89**	**1.32**
YOLOv8-Ghost				
A1	**83.67**	96.46	**0.67**	**1.21**
A2	**83.30**	93.89	0.78	1.00
A3	69.49	92.00	−1.18	1.43

The result in bold font means that the result is superior to others.

**Table 7:** Results and rankings on the leaderboard (on 20 April 2024).

Test Set	BD	FGBGDice	DiffFG	AbsDiffFG
A1	85.18 (5th)	96.46 (5rd)	0.67 (6th)	1.21 (4th)
A2	84.03 (4th)	93.89 (4th)	0.78 (7th)	1.00 (2nd)
A3	86.11 (1st)	92.00 (4th)	−1.18 (4th)	1.43 (1st)
A4	86.58 (4th)	95.44 (4th)	0.79 (8th)	1.11 (3rd)
A5	86.52 (4th)	94.61 (3rd)	0.29 (8th)	1.17 (3rd)
ALL	86.36 (4th)	94.70 (3rd)	0.32 (8th)	1.18 (3rd)

## Data Availability

The data presented in this study are available in GitHub repository: https://github.com/rexlagrange/cvppp_leaf_seg, accessed on 1 June 2024. These data were derived from the following resources available in the public domain: https://codalab.lisn.upsaclay.fr/competitions/8970, accessed on 1 January 2024.
